# A systematic review and meta-analysis of paraoxonase-1 activity in asthma

**DOI:** 10.1007/s10238-022-00930-0

**Published:** 2022-11-07

**Authors:** Stefania Bassu, Arduino A. Mangoni, Dario Argiolas, Ciriaco Carru, Pietro Pirina, Alessandro G. Fois, Angelo Zinellu

**Affiliations:** 1 Department of Biomedical Sciences, University of Sassari, Viale San Pietro 43, 07100 Sassari, Italy; 2 Discipline of Clinical Pharmacology, College of Medicine and Public Health, Flinders University, Adelaide, Australia; 3 Department of Clinical Pharmacology, Flinders Medical Centre, Southern Adelaide Local Health Network, Adelaide, Australia; 4 Department of Clinical, Surgical and Experimental Medicine, University of Sassari, Sassari, Italy

**Keywords:** Disease, Inflammation, PON-1, Oxidative stress, Chronic

## Abstract

Human serum paraoxonase-1 (PON-1) is a critical antioxidant defence system against lipid oxidation. Decreased PON-1 activity has been associated with systemic oxidative stress in several disease states. We conducted a systematic review and meta-analysis of plasma/serum concentrations of PON-1 in asthma, a chronic inflammatory airway disease. The electronic databases PubMed, Web of Science, Scopus and Google Scholar were searched from inception to February 2022. In total, 8 studies in 355 asthmatic patients and 289 healthy controls were included in the meta-analysis. Serum PON-1 concentrations were significantly lower in asthmatic patients (SMD = −1.58, 95% CI −2.53 to −0.63; *p* = 0.001). The pooled SMD values were not substantially altered in sensitivity analysis. There was no publication bias. There were non-significant differences in PON-1 concentrations in patients with severe vs. mild-to-moderate asthma (SMD =  − 0.39, 95% CI − 1.00 to 0.22, *p* = 0.21). Our meta-analysis has shown that serum PON-1 concentrations are significantly lower in patients with asthma, suggesting the presence of an impaired antioxidant defense in this group.

## Introduction

Asthma is a chronic airway disease that is characterized by chronic inflammation with activation of inflammatory cells and mediators, airway hyper-responsiveness, excess mucus production and epithelial cell shedding [1]. Macrophages, neutrophils, and eosinophils infiltrate the bronchial mucosa in asthmatic patients, releasing reactive oxygen species (ROS) [2]. Previous studies have demonstrated the association between chronic inflammation and oxidative stress. Elevated concentrations of ROS, e.g., hydroxyl radicals, superoxides, and peroxides can lead to increased airway reactivity and secretions, and increased vascular permeability, which collectively augment the inflammatory burden, a hallmark of asthma [3]. Increased production of ROS, leading to an imbalance between pro- and antioxidant defence systems, causes local and systemic oxidative injury in asthmatic patients [4, 5].

Human serum paraoxonase-1 (PON-1), an antioxidant and anti-inflammatory calcium-dependent esterase associated with high-density lipoprotein (HDL), used as a marker of lipid peroxidation, prevents the oxidation of low-density lipoproteins (LDL) and the consequent generation of lipid peroxides [6–8]. PON-1 is primarily expressed in the liver and released in the blood with the HDL particle [9]. Serum PON-1 hydrolyzes pro-inflammatory oxidized lipids, typically presenting as ox-LDL, and suppresses their atherogenic effects [10].

Decreased PON-1 activity is a marker of increased systemic oxidative stress and increased conversion of HDL to a dysfunctional pro-inflammatory and pro-atherogenic state. Not surprisingly, decreased PON-1 activity has been associated with the development of several diseases such as atherosclerosis [11, 12], psoriasis [13], Sjögren's syndrome [14], and rheumatoid arthritis [15]. Associations between reduced PON-1 concentrations and respiratory disease states such as obstructive sleep apnea and chronic obstructive pulmonary disease have also been described [16, 17].

In order to capture and interpret the available evidence regarding the relationship between PON-1 activity and asthma, we conducted a systematic review and meta-analysis of studies reporting plasma/serum concentrations of PON-1 activity in asthmatic patients and control groups.

## Methods

### Search strategy, eligibility criteria, and study selection

We conducted a systematic review of published studies in the electronic databases Pubmed, Web of Science, Scopus and Google Scholar from inception to February 2022. The terms “paraoxonase” or “PON” or “paraoxonase-1” or “PON-1” or “asthma” and their combinations were used for the search. Moreover, references of individual studies were manually checked to identify additional studies. Data extraction and quality assessment were performed independently by two investigators. Eligibility criteria were: (i) assessment of paraoxonase activity in plasma or serum evaluated as rate of paraoxon hydrolysis; (ii) comparison of patients with stable asthma and controls (case–control design); (iii) sample size ≥ 10 patients; (iv) English language and (v) full-text publications.

The Joanna Briggs Institute (JBI) Critical Appraisal Checklist for analytical studies was used to assess the risk of bias. A score of ≥ 5, 4, and < 4 indicated low, moderate, and high risk, respectively [18]. The certainty of evidence was assessed using the Grades of Recommendation, Assessment, Development and Evaluation (GRADE) Working Group system. GRADE considers the study design (randomized vs. observational), the risk of bias (JBI checklist), the presence of unexplained heterogeneity, the indirectness of evidence, the imprecision of results (sample size, 95% confidence interval width and threshold crossing), the effect size (small, SMD < 0.5, moderate, SMD 0.5–0.8, and large, SMD > 0.8) [19], and the probability of publication bias [20, 21]. The study complied with the Preferred Reporting Items for Systematic reviews and Meta-Analyses (PRISMA) 2020 statement [22].

### Statistical analysis

Standardized mean differences (SMD) with 95% confidence intervals (CIs) were used to create forest plots of continuous data and to evaluate differences in PON-1 activity between asthmatic patients and controls (*P* < 0.05 for statistical significance). If necessary, means and standard deviations were extrapolated from medians and interquartile ranges, as previously reported by Wan et al. [23].

As suggested in the Cochrane handbook (24), we treated *I*^2^ < 30% as no or slight heterogeneity; otherwise, there was moderate or substantial heterogeneity (*I*^2^ ≥ 30%). For meta-analyses with moderate or substantial heterogeneity a random-effect model based on the inverse-variance method was conducted [24, 25].

The influence of individual studies on the effect size was evaluated by their sequential exclusion through sensitivity analysis [26]. Publication bias was assessed by means of Begg’s adjusted rank correlation test and Egger’s regression asymmetry test (*p* < 0.05 for statistical significance) [27, 28]. Statistical analyses were performed using Stata 14 (STATA Corp., College Station, TX, USA). The study protocol was registered in the International Prospective Register of Systematic Review (PROSPERO) registration number CRD42022316757.

## Results

### Systematic research and study characteristics

**Fig. 1:**
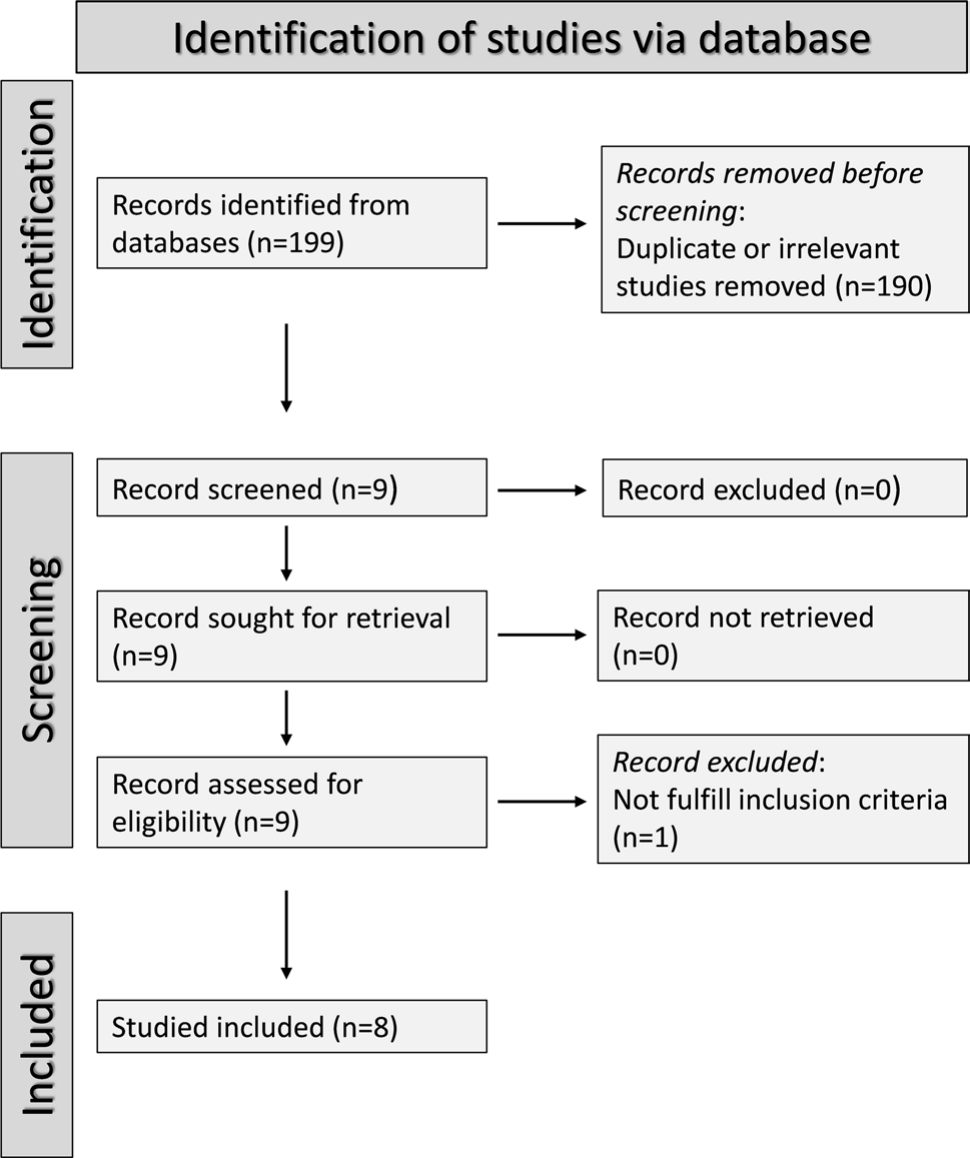
PRISMA 2020 flow diagram

A flow chart describing the screening process is presented in Fig. 1. We initially identified 199 studies. A total of 190 were excluded after the first screening because they were either duplicates or irrelevant. After a full-text revision of the remaining 9 articles, one was excluded because it did not fulfil the inclusion criteria, leaving 8 studies for the final analysis [29–36]. There was no disagreement between the two independent investigators. A total of 355 patients with a mean age of 23 years (55% males) and 289 healthy controls with a mean age of 28 years (61% males) were assessed. The characteristics of the retrieved studies, published between 2004 and 2018, are reported in Table 1.

**Table 1:** Study characteristics

First Author and Year, Country	Controls				Asthma				
	N	Age Mean or median	M/F	PON Mean ± SD (U/L)	N	Age Mean or median	M/F	PON Mean ± SD (U/L)	FEV_1_ (%)
Ekmekci et al. 2006, Turkey	28	36	NR	160 ± 68	30	36	NR	135 ± 68	78
Can et al. 2007, Turkey	25	10	14/11	231 ± 109	25	9	11/14	211 ± 123	86
Cakmak et al. 2009, Turkey	32	9	10/22	349 ± 154	42	9	20/22	164 ± 73	NR
Acay et al. 2013, Turkey	20	44	16/4	521 ± 126	40	39	16/24	65 ± 52	NR
Emin et al. 2015, Turkey	55	11	35/20	297 ± 88	85	10	50/35	157 ± 55	81
Sarioglu et al. 2015, Turkey	41	45	34/7	237 ± 174	49	46	37/12	164 ± 114	90
Zinellu et al. 2016, Italy	24	60	11/13	245 ± 124	24	59	11/13	242 ± 184	NR
EL-Alameey et al. 2019, Egypt	60	NR	NR	212 ± 19	60	8	NR	143 ± 19	73

NR: Not Reported

### Risk of bias

The risk of bias was considered low in all studies (Table 2).

**Table 2:** The Joanna Briggs Institute critical appraisal checklist

Study	Were the criteria for inclusion in the sample clearly defined?	Were the study subjects and the setting described in detail?	Was the exposure measured in a valid and reliable way?	Were objective, standard criteria used for measurement of the condition?	Were confounding factors identified?	Were strategies to deal with confounding factors stated?	Were the outcomes measured in a valid and reliable way?	Was appropriate statistical analysis used?	Risk of bias
Ekmekci et al	Yes	Yes	Yes	Yes	No	No	Yes	No	Low
Can et al	Yes	Yes	Yes	Yes	No	No	Yes	No	Low
Cakmak et al	Yes	Yes	Yes	Yes	No	No	Yes	No	Low
Acay et al	Yes	Yes	Yes	Yes	Yes	Yes	Yes	Yes	Low
Emin et al	Yes	Yes	Yes	Yes	No	No	Yes	No	Low
Sarioglu et al	Yes	Yes	Yes	Yes	No	No	Yes	No	Low
Zinellu et al	Yes	Yes	Yes	Yes	Yes	Yes	Yes	Yes	Low
EL-Alameey et al	Yes	Yes	Yes	Yes	Yes	Yes	Yes	Yes	Low

### Results of individual studies and syntheses

**Fig. 2:**
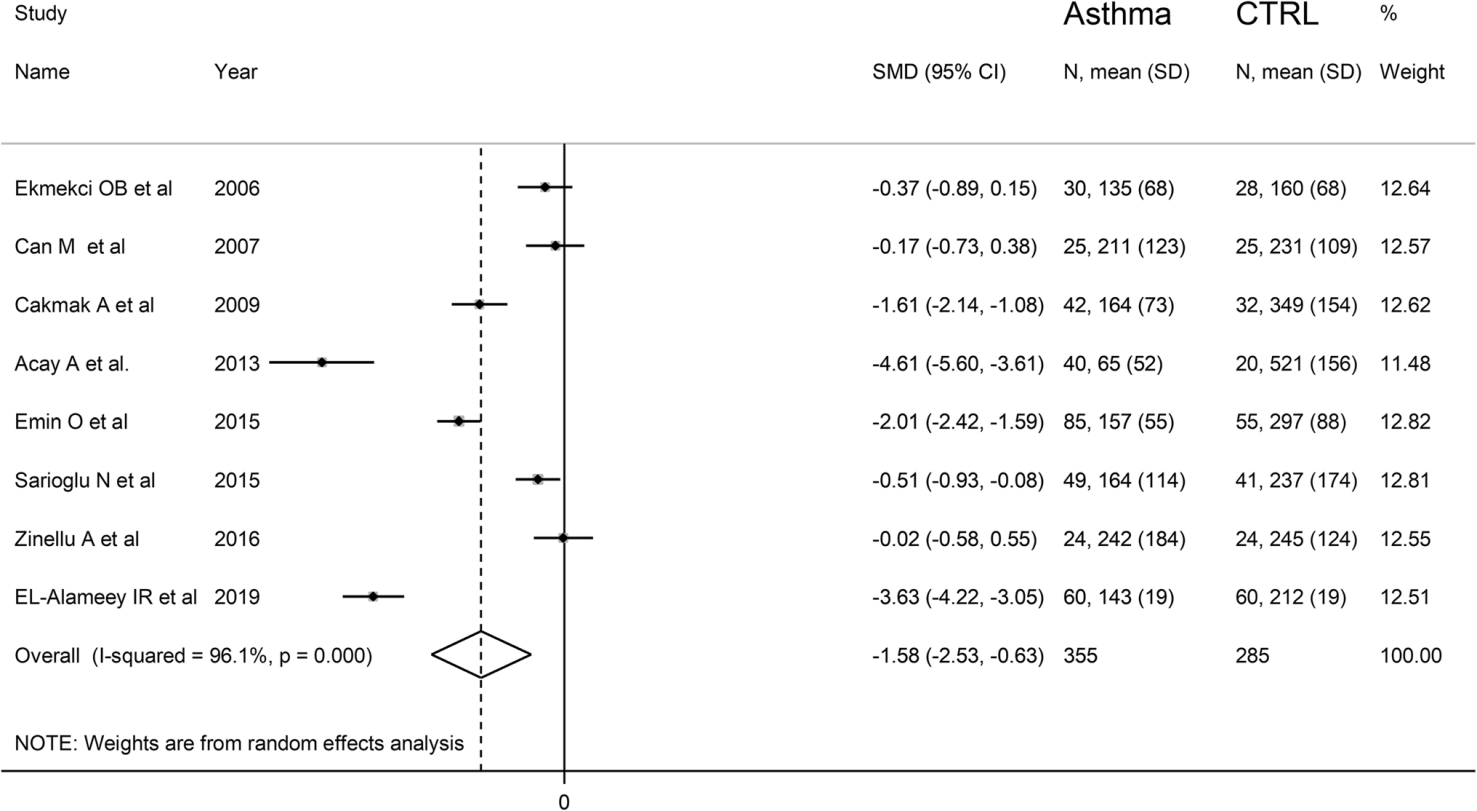
Forest plot of studies examining serum PON concentrations in asthmatics and controls

**Fig. 3:**
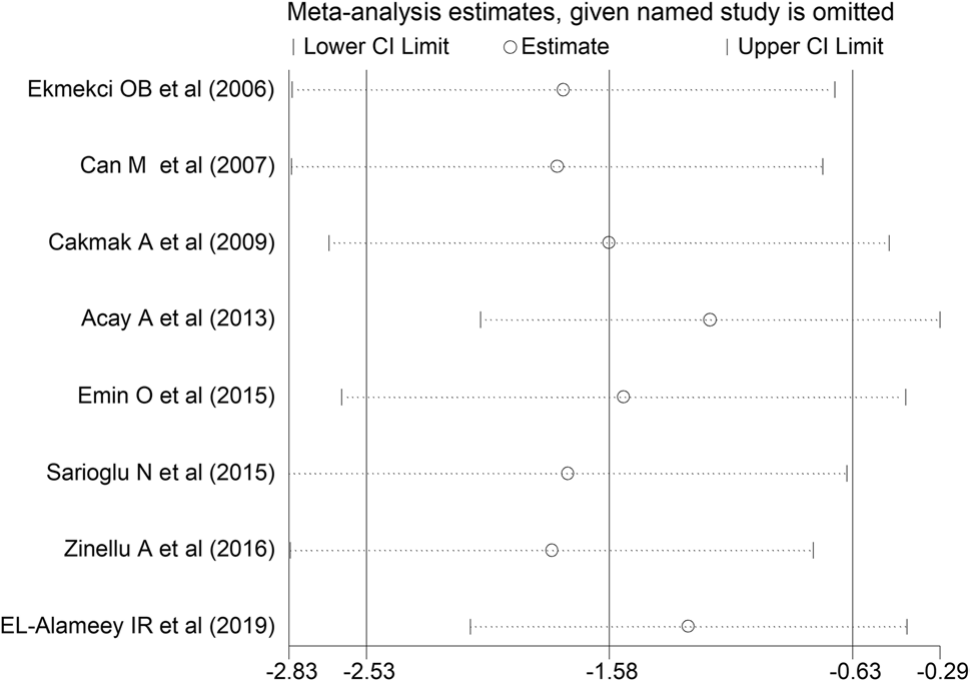
Sensitivity analysis of the association between serum PON values and asthma. For each study, the displayed effect size (hollow circles) corresponds to an overall effect size computed from a meta-analysis excluding that study

The forest plot for PON-1 activity in asthmatic patients and controls is reported in Fig. 2. In all studies, asthmatic patients had lower PON-1 activity than controls (mean difference range, −0.02 to −4.61) and the difference was statistically significant in five studies [31–34, 36]. Substantial heterogeneity between studies was observed (*I*^2^ = 96.1%, *p* < 0.001). Thus, random-effects models were used. Overall, pooled results showed that PON-1 activity was significantly lower in asthmatic patients (SMD = −1.58, 95% CI −2.53 to −0.63; *p* = 0.001). In sensitivity analysis, the corresponding pooled SMD values were not altered when individual studies were sequentially omitted (effect size range, between −1.80 and −1.18, Fig. 3).

### Publication bias

**Fig. 4:**
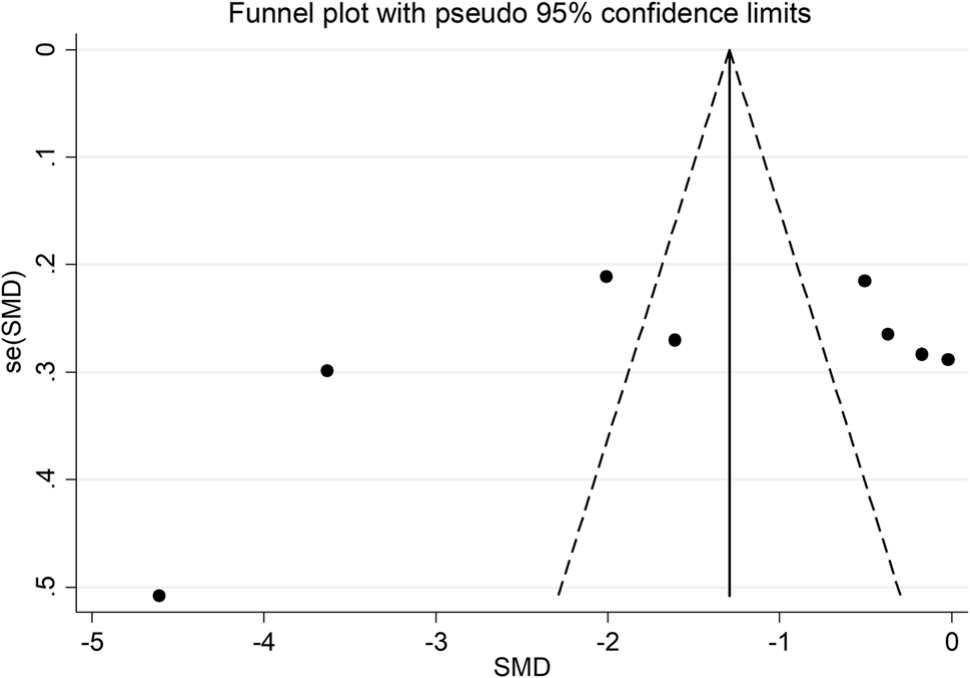
Funnel plot of studies investigating the association between serum PON concentrations and asthma

Bias analysis indicated the absence of publication bias (Begg’s test, *p* = 0.54; Egger’s test, *p* = 0.33). However, the funnel plot analysis, reported in Fig. 4, detected a distortive effect of two studies [32, 36]. After removing these studies, the effect size was attenuated but remained significant (SMD =  − 0.79, 95% CI − 1.47 to − 0.10, *p* = 0.02, *I*^2^ = 91.5%, *p* < 0.001).

### Sub-group analysis

**Fig. 5:**
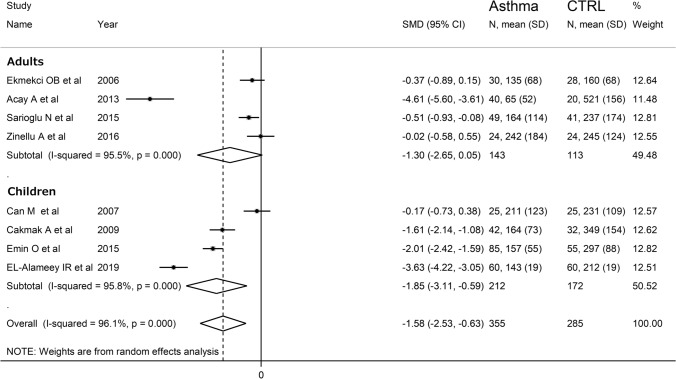
Forest plot of studies examining serum PON concentrations of asthmatics and controls according to age

**Fig. 6:**
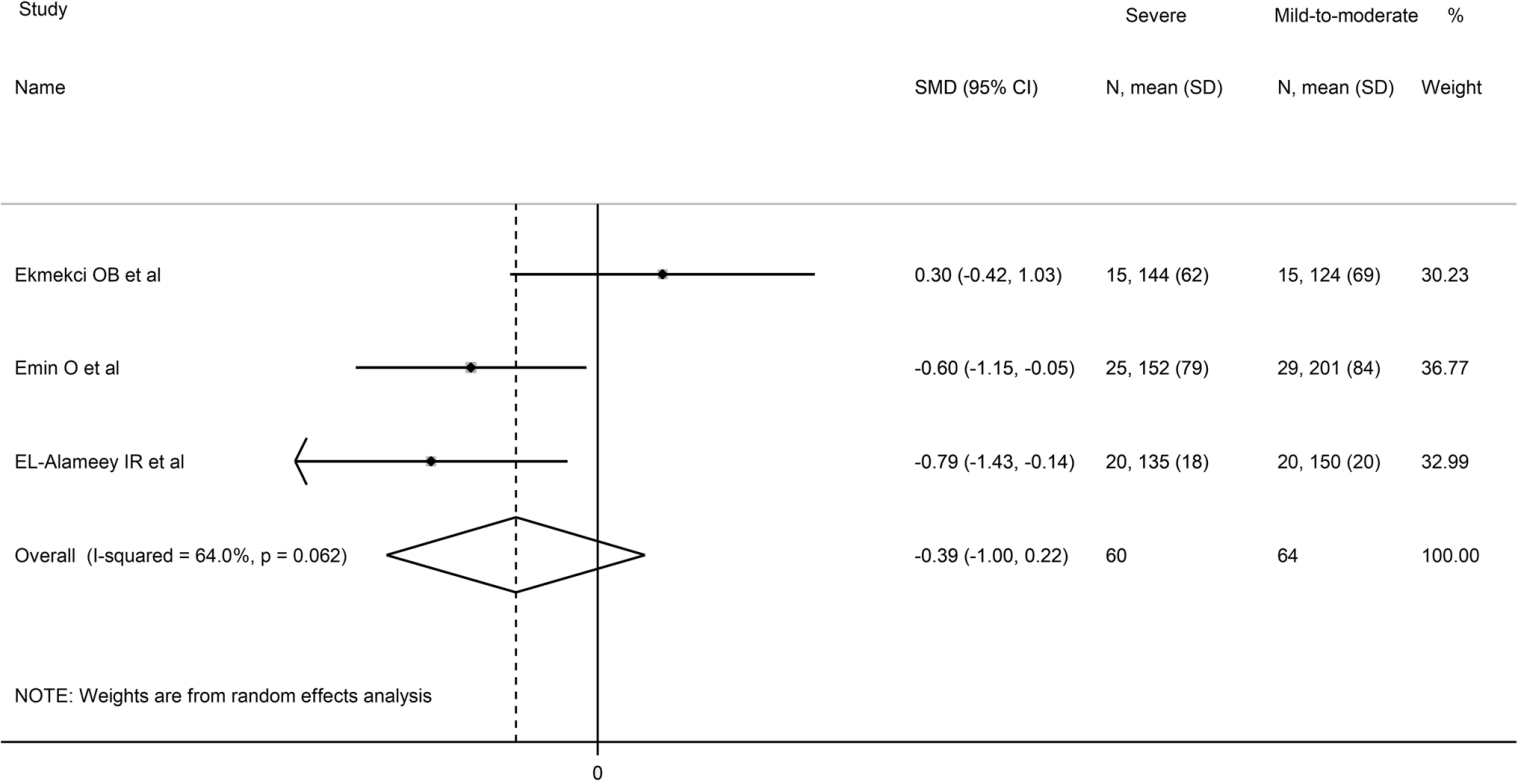
Forest plot of studies examining serum PON concentrations of asthmatic patients according to disease severity

Sub-group analysis showed that asthma-associated PON-1 reduction was significant in children (SMD = −1.85, 95% CI 3.11 to −0.59, *p* = 0.004) but not in adults (SMD = −1.30, 95% CI −2.65 to 0.05, *p* = 0.06) (Fig. 5). Heterogeneity remained extreme in both groups (95.8% and 95.5%, respectively). Three studies also reported PON-1 data in relation with disease severity. As shown in Fig. 6, there were non-significant differences in serum PON-1 activity between severe and mild-to-moderate asthmatics (SMD =  − 0.39, 95% CI − 1.00 to 0.22, *p* = 0.21, *I*^2^ = 64.0%, *p* = 0.062).

### Certainty of evidence

The initial level of certainty for PON-1 SMD values was considered low because of the cross-sectional nature of the studies (rating 2, ⊕  ⊕ ⊝ ⊝). After considering the low risk of bias in all studies (no rating change required), the extreme and unexplained heterogeneity (downgrade one level), the lack of indirectness (no rating change required), the relatively low imprecision (relatively narrow confidence intervals without threshold crossing, no rating change required), the large effect size (SMD = −1.58, upgrade one level), and the absence of publication bias (no rating change required), the overall level of certainty remained low (rating 2, ⊕  ⊕ ⊝ ⊝).

## Discussion

In our systematic review and meta-analysis, plasma/serum PON-1 activity was significantly lower in asthmatic patients when compared to healthy controls. The relatively large SMD values for PON-1 activity indicate an effect size that is likely to be of biological and/or clinical relevance. Despite the substantial heterogeneity observed, in sensitivity analysis the effect size of PON-1 activity was not significantly affected when each study was in turn removed. The studies by Acay et al. [32] and El-Alameey et al. [36] influenced graph symmetry with a possible effect on the effect size. After removing these studies, the SMD remained significant with persistent substantial heterogeneity between studies. Further analyses based on the Begg's and Egger's t-tests did not show publication bias. In sub-group analysis, asthmatic children, but not adults, showed significantly lower PON-1 activity vs. control groups. This is in line with previous studies reporting that asthmatic children have higher levels of oxidative stress than asthmatic adults [37, 38]. Whilst the exact factors involved remain elusive, Cakmak et al. [31] suggested that the challenges associated with successfully managing asthma in some pediatric patients might lead to increased oxidative stress.

Although Gornicka et al. [39] showed that lower PON-1 activity may be also related to disease severity, our pooled data did not demonstrate significant differences in serum PON-1 activity between severe and mild-to-moderate asthmatics. However, it should be emphasized that our results have been extrapolated by pooling only three studies, therefore additional research is warranted to address this issue. It is important to point out that differences in PON-1 activity may also be related to geographical and/or ethnic factors [29].

In addition, several PON-1 gene polymorphisms may affect PON-1 activity, particularly Q192R and L55 polymorphisms [29]. Polonikov et al. [40] reported that the PON-1 QR gene polymorphism was significantly associated with the risk of asthma. In contrast, Tölgyesi et al. [41] reported that PON-1 polymorphisms did not significantly influence the susceptibility to asthma. As the selected studies did not investigate the role of PON-1 gene polymorphisms, further research is warranted to investigate interplay between specific PON-l gene polymorphisms, PON-l activity, and asthma.

Although the exact mechanisms responsible for the lower serum activity of PON-1 activity in asthma are unclear, the imbalance between excessive production of free radicals and deficiency in antioxidant defence system might play an important role [42]. PON-1 activity reduction can be related to increased lipid peroxidation, caused by ROS produced by inflammatory cells, as oxidized lipids are suggested to block PON-1 activity [43]. A reduced PON-1 activity has been also observed in other chronic inflammatory disease states. For example, in a cohort of patients with atherosclerosis, those with the lowest PON-1 activity had a 3.4 times greater hazard of major cardiovascular events compared to those with the highest PON-1 activity [44]. Impaired PON-1 activity has also been reported to be associated with a higher prevalence of atherosclerotic cardiovascular disease in patients with rheumatoid arthritis and Alzheimer’s disease [45, 46]. Circulating concentrations of leptin, hs-CRP and IL-6 have been found to be significantly associated with PON-1 activity [47]. Furthermore, there is good evidence that PON-1 protects lipids against peroxidation by preventing low-density lipoprotein oxidation, a critical factor involved in the pathogenesis of inflammatory diseases such as atherosclerosis, diabetes, and cancer [48].

Limitations of our study include the substantial between-study heterogeneity and the lack of meta-regression analyses to identify parameters responsible for the between-study variance, due to the small number of articles. The lack of information provided in the selected studies on PON-l gene polymorphisms, body mass index (associated with the risk of asthma), and use of specific drugs (e.g., metformin, associated with increases PON-1 activity) represent additional limitations of our study. However, there was no evidence of publication bias, and the overall effect size was not significantly influenced in sensitivity analyses. In addition, the comprehensive evaluation of the risk of bias and the certainty of evidence according to GRADE, and the demonstration that arylesterase activity (not associated with PON-1 polymorphism) was also decreased in asthma patients significantly strengthen the conclusion of our study.

## Conclusion

Our systematic review and meta-analysis have shown that serum activity of PON-1 is significantly lower in asthmatic patients. Additional prospective studies are required to investigate the clinical impact of PON-1 activity in this group.
